# Designing for Risk Assessment Systems for Patient Triage in Primary Health Care: A Literature Review

**DOI:** 10.2196/humanfactors.5083

**Published:** 2016-08-15

**Authors:** Alessandro Jatoba, Catherine Marie Burns, Mario Cesar Rodriguez Vidal, Paulo Victor Rodrigues Carvalho

**Affiliations:** ^1^ Fundação Oswaldo Cruz Centro de Estudos Estratégicos Rio de Janeiro Brazil; ^2^ University of Waterloo Department of Systems Design Engineering Waterloo, ON Canada; ^3^ Universidade Federal do Rio de Janeiro COPPE Rio de Janeiro Brazil; ^4^ Instituto de Engenharia Nuclear Rio de Janeiro Brazil

**Keywords:** primary health care, triage, clinical decision support systems, health information systems

## Abstract

**Background:**

This literature review covers original journal papers published between 2011 and 2015. These papers review the current status of research on the application of human factors and ergonomics in risk assessment systems’ design to cope with the complexity, singularity, and danger in patient triage in primary health care.

**Objective:**

This paper presents a systematic literature review that aims to identify, analyze, and interpret the application of available evidence from human factors and ergonomics to the design of tools, devices, and work processes to support risk assessment in the context of health care.

**Methods:**

Electronic search was performed on 7 bibliographic databases of health sciences, engineering, and computer sciences disciplines. The quality and suitability of primary studies were evaluated, and selected papers were classified according to 4 classes of outcomes.

**Results:**

A total of 1845 papers were retrieved by the initial search, culminating in 16 selected for data extraction after the application of inclusion and exclusion criteria and quality and suitability evaluation.

**Conclusions:**

Results point out that the study of the implications of the lack of understanding about real work performance in designing for risk assessment in health care is very specific, little explored, and mostly focused on the development of tools.

## Introduction

In health care, patient triage and risk assessment has always been a major concern [1-4]. Keeping patients safe and ensuring that they receive the right treatment has been studied in different research areas such as psychology [5,6], software engineering [7,8], ergonomics [9-11], and others. These studies of how health care workers make decisions in such complex systems have given some insights into how to design for patient safety.

Furthermore, in order to improve patient triage, system designers must understand functional work requirements and constraints in the beginning of the design process; otherwise, it becomes difficult to incorporate human factors after the design is completed [12]. While interacting with a complex physical environment, only a few elements of a problem can be within the span of human conscious attention simultaneously [13]. Moreover, different levels of complexity exist, and it is virtually impossible to reduce the number of variables of a complex system without losing its essential properties [14].

Thus, the objective of this paper was to present a systematic literature review that aimed to identify, analyze, and interpret available scientific evidence related to the contributions of cognitive engineering [15,16] to the design of tools, devices, and work processes to support patient triage and risk assessment. This paper reviews the state-of-the-art research in this theme, identifying gaps in order to suggest further investigation. We explore the topic of decision-making in patient triage, examining the extent to which empirical evidence supports or contradicts the theoretical hypothesis that formative approaches, such as those commonly included in cognitive engineering approaches, are important for the design for the health care domain.

The conceptual significance of this paper resides in providing the means to help researchers understand how the disciplines of ergonomics and human factors contribute to the improvement of work situations in health care, enhancing the design of devices and work processes to support effective behaviors [17] in the patient triage and risk assessment process.

## Methods

### Databases and Search

The authors performed an electronic search on 7 bibliographic databases: ScienceDirect, PubMed, SpringerLink, ACM Digital Library, Wiley Online Library, Scopus, and IEEE Xplore.

We considered these databases appropriate because of the quantity of indexed journals and coverage of relevant disciplines such as health sciences, engineering, and computer sciences. The flexibility of their search engines (for combining search terms) and the ability to export results to formats accepted by reference managing software were also considered in the selection of academic databases.

### Research Question

In this literature review we collected, classified, and analyzed recent work related to the topic of risk assessment in health care. We have highlighted scientific evidence on the efforts that have been made to improve the design of technology, medical devices, tools, and processes, to support decision making in patient risk assessment. The following research question motivated this review: What are the contributions, advantages, and disadvantages of using cognitive engineering in the design of software for risk assessment during patient triage?

### Selection Criteria

This literature review included original journal papers published in English between 2011 and 2015, including papers available online in 2015. This time frame was chosen in order to concentrate on more recent contributions and represent the current status of research related to our topic. Conference papers, books, chapters, and reports have not been included in this literature review.

Table 1 presents a summary of the search terms and respective variations derived from the research question. We have used free search terms with no controlled descriptors in order to have a broader search.

**Table 1:** Search terms and variations.

Term	Variations
Cognitive engineering	Cognitive ergonomics; cognitive systems engineering; cognitive work analysis; cognitive task analysis; human factors; ergonomics
Risk assessment	Triage; patient triage; risk management
Health care	Medical care; clinical care; emergency care

We used variations of search terms to match eventual synonyms, abbreviations, alternative spellings, and related topics. The authors performed trial searches using various combinations of search terms in order to check the search terms against lists of already known primary studies, using the following search query: (“Human factors” OR “Ergonomics” OR “Cognitive ergonomics” OR “Cognitive engineering” OR “Cognitive systems engineering” OR “Cognitive work analysis” OR “Cognitive task analysis”) AND (“Risk assessment” OR “Triage“ OR ”Patient triage” OR “Risk management”) AND (“Health care” OR “Medical care” OR “Clinical care” OR “Emergency care”).

We describe inclusion and exclusion criteria in Textbox 1.

**Inclusion and exclusion criteria.**

Inclusion criteria

- Studies that assess difficulties, critical factors, challenges, or problems in applying human factors and ergonomics in the design of risk assessment support tools or processes in health care
- Studies that present good practices, lessons learned, and success factors in applying human factors and ergonomics concepts in the design of systems for patient triage and risk assessment
- Studies presenting models, processes, techniques, or tools to enable the improvement of patient triage and risk assessment in health care

Exclusion criteria

- Studies that do not address any of the research questions
- Literature reviews

In addition to general inclusion and exclusion criteria, the quality of primary studies has been evaluated, as well as their suitability to the presented research questions, in order to investigate whether quality differences provide useful explanations, guide the interpretation of findings, and determine the strength of inferences, as well as how they address the research questions. The quality of a scientific study relates to the extent to which it minimizes bias and maximizes internal and external validity [18]. The following aspects have been evaluated in the study:

- The objective, research questions, and methods are well defined
- The contributions are well described
- The kind of scientific study is clearly stated
- The source population is identified
- The interventions or strategies are sufficiently described to allow reasonable replication
- The outcome is defined and measurable
- The objectives are accomplished and research questions are clearly answered
- The study addresses the research question

Selected publications were given scores from 1 to 5 for each aspect, where 1 corresponds to “strongly disagree” and 5 “strongly agree.” The sum of the scores determined their methodological quality and suitability to research question as follows:

- Very high, 100% of the methodological quality aspects met
- High, 75%-99% met
- Medium, 50%-74% met
- Low, 0%-49% met

A committee of 4 researchers applied the inclusion and exclusion criteria and performed the assessment of methodological quality of the selected papers. Committee members were doctorate students in systems design engineering and had similar levels of expertise in ergonomics and human factors. A tenured professor, head of the ergonomics and human factors laboratory, supervised the committee during the process. After reading the papers, the committee met in order to present their evaluation. The final score for each criterion for methodological quality represents the consensus of committee members. A study proceeded to data extraction when it met a score of at least 50% on methodological quality.

### Definition of Outcomes

We stratified the selected papers according to 4 classes of outcomes as follows:

- Class A—design of risk assessment decision support for health care: papers fit this class when the outcomes proposed the implementation of new tools to support decision making in health care risk assessment work situations;
- Class B—design frameworks, processes, and methods for risk assessment in health care: this class related to publications where outcomes presented frameworks or processes applied to the design of risk assessment work situations in health care environments;
- Class C—recommendation or implementation of improvements in risk assessment work situations in health care: this class of outcomes was met by papers that suggested transformations in the work place, environment, equipment, or processes in risk assessment work situations in health care;
- Class D—analysis of the effect of new technology or processes to risk assessment in health care: this class was met by papers that presented studies about the implications of transformations made by new devices or processes for risk assessment in health care environments.

Papers selected for data extractions were also classified according to the type of study: case study, experimental study, exploratory study, empirical study, or field study.

## Results

### Outcome Statistics

Among the 7 databases searched, 5 of them had their results exported to a library in the reference management software Zotero (Roy Rosenzweig Center for History and New Media, George Mason University). The results of 2 of them (IEEE Xplore and SpringerLink) could not be exported to Zotero because of limitations of the search engine but could be exported in CSV format and organized in Microsoft Excel spreadsheets. The steps for paper selection included reading the title, abstract, and full paper. Exclusions on the first and second steps were based on how titles and abstracts of papers indicated relations with the topic we explored in this literature review [18-20]. On the third step, inclusion and exclusion criteria were applied in order to select papers for data extraction. Table 2 presents the results of paper selection steps and the distribution of the papers across the various databases.

**Table 2:** Summary of search results.

Database	Selected papers				
	Search results, N	Selected after title reading	Selected after abstract reading	Selected after full reading, n	Percentage of selected papers, %
ScienceDirect	403	55	8	4	1.0
PubMed	249	19	6	5	2.0
SpringerLink	149	27	3	2	1.3
ACM Digital Library	159	18	3	2	1.3
Wiley Online Library	238	22	5	1	0.4
Scopus	33	10	5	1	3.0
IEEE Xplore	614	31	6	1	0.2
Total	1845	182	36	16	0.9

We retrieved 1845 papers in the initial search. After reading the titles and abstracts 36 papers were selected for full reading. Among these, 16 papers met the inclusion and exclusion criteria and were submitted to quality and suitability evaluation, as well as data extraction. Table 3 summarizes the key elements of these selected papers. The outcome code refers to the outcome categories that were defined in the Definition of Outcomes subsection. All papers listed in Table 3 reached 50% or more on the score for methodological quality.

Most of the studies are case studies (8 papers), followed by exploratory studies (6 papers). Finally, 2 of the 16 selected papers are experimental studies. We proceeded with the data extraction and the stratification of papers according to the 4 classes of outcomes described in the Definition of Outcomes subsection and listed in the Outcome column of Table 3. In Table 4, the distribution of these outcome types, across the various databases, is presented. The final distribution of papers by the databases was examined as it gives some guidance in terms of where future researchers may wish to look for relevant high-quality papers in the human factors and ergonomics approaches to health care.

**Table 3:** Summary of selected papers.

Authors	Summary	Type of study	Outcome
McClean et al [26]	McClean et al propose the use of a framework for modeling the care process in hospitals in order to improve the assessment of patients’ clinical status and define the length of their stay at the hospital. The paper presents a case study based on data extracted from patients of a hospital in Belfast and demonstrates results of patient survival rates when using their length of stay and destination as outcomes.	Case study	B
Alemdar et al [24]	The authors adopt techniques for human behavior analysis from a medical perspective through the analysis of daily activities in terms of timing, duration, and frequency and propose an evaluation method applicable to real-world applications that require human behavior understanding through an experimental study.	Experimental study	A
Hundt et al [25]	According to Hundt et al most vulnerability in the design of computerized tools to support physician order entry occur by not considering the work system in which the technology is implemented; therefore, the authors state that the human factors engineering discipline offers a range of approaches for anticipating vulnerabilities, enabling designers to address them before technology implementation.	Case study	A
Card et al [27]	Card et al present a case study that shows the rationale for taking a proactive approach to improving health care organizations’ emergency operations. It demonstrates how the Prospective Hazard Analysis Toolkit can drive organizational learning and improve work situations.	Case study	B
Pennathur et al [28]	Through a study conducted in hospitals, Pennathur et al propose an information trail model for capturing fundamental characteristics of information that workers in emergency departments create and use for patient care. The model proposed by Pennathur et al addresses our research subquestions by presenting a method for tackling complexity and prevents failures by increasing understanding of the information flow in the process of assessing patient conditions, based on the idea that people in a complex cognitive work system organize information on their own.	Exploratory study	B
Aringhieri et al [30]	In their paper, Aringhieri et al present an exploratory study on the ambulance location and management in the Milano area, in which they evaluate the current emergency system performance. According to the authors, despite the availability of technological support, in Italy, the use of resources in emergency departments is based on operators’ experience.	Exploratory study	C
Iakovidis and Papageorgiou [22]	Iakovidis and Papageorgiou propose a model and evaluate its effectiveness in two scenarios for pneumonia risk assessment. Their results indicate that the major contribution of the proposed model is that it incorporates additional information regarding the hesitancy of the experts in the definition of the cause-effect relations between the concepts involved in the health care domain. Iakovidis and Papageorgiou state that the proposed approach is capable of modeling real-world medical decision-making tasks closer to the way humans perceive them.	Exploratory study	A
Kong et al [23]	Kong et al propose the employment of a belief rule-base inference methodology using the evidential reasoning approach in order to support modeling and reasoning with clinical domain knowledge. According to Kong et al, the approach they propose helps in reducing uncertainties in clinical signs, clinical symptoms, and clinical domain knowledge, which are critical factors in medical decision making.	Exploratory study	A
Cagliano et al [29]	Cagliano et al propose a framework that operationalizes Reason’s theory of failures [42] by developing a methodology for investigating health care processes and related risks in patients based on expert knowledge. They apply their approach to the pharmacy department of a large hospital.	Exploratory study	B
Park et al [39]	Park et al studied how the design of electronic medical record (EMR) systems affects medical work practices. They analyzed consequences of EMR on clinical work practices and related design issues, such as usability or functionalities of EMR systems, in order to associate the work practices changes led by the EMR system with the actual design of the system.	Case study	D
Hepgul et al [31]	Hepgul et al present an examination of the role of clinical expertise and multidisciplinary teams in identifying patients at risk of developing depression, and in monitoring those receiving treatment for the occurrence of depression.	Case study	C
Glasgow et al [40]	Glasgow et al propose a comparison between risk estimates from statistical models previously developed and evaluated and risk estimates from the patients’ surgeons. Through this comparison, they are able to evaluate the predictive validity of the decision support model for safer surgery in predicting risk for specific complications. Moreover, they enable the assessment of the validity of this model by correlating its predictions to the ones made by experienced surgeons.	Exploratory study	D
Johnston et al [32]	Johnston et al describe the importance of overcoming hierarchical barriers between junior and senior surgeons as a crucial success factor for prioritization of health care.	Case study	C
Ferguson and Starmer [35]	Ferguson and Starmer highlight the role of expertise in risk assessment in health care facilities and evaluate the effects of framing risks on the improvement of interpretation in such environments.	Experimental study	C
Norris et al [33]	In their paper, Norris et al describe a project that takes a systems approach to identify risks, engage health care staff and patients, facilitate ideas, and develop new designs for the bed-space in order to demonstrate the application of human factors to a complete design cycle.	Case study	C
Hastings et al [34]	Hastings et al propose a method to classify older adults in the emergency department according to health care use, by examining associations between group membership and future hospital admissions.	Case study	C

**Table 4:** Publications classified according to outcomes, distributed by databases.

Database	Outcomes			
	A Design of risk assessment decision support for health care	B Design frameworks, processes, and methods for risk assessment in health care	C Recommendation or implementation of improvements in risk assessment work situations in health care	D Analysis of the effects of new technologies or processes to risk assessment in health care
ScienceDirect	1	1	1	1
PubMed	-	-	4	1
SpringerLink	-	1	1	-
ACM Digital Library	1	1	-	-
Wiley Online Library	-	1	-	-
Scopus	1	-	-	-
IEEE Xplore	1	-	-	-
Total	4	4	6	2
Percentage, %	25	25	38	12

In the next subsections, we present an overview of the selected publications, describing how they address our research questions.

### Design of Risk Assessment Decision Support for Health Care

Cognitive ergonomics is concerned with mental processes, such as perception, memory, reasoning, and motor response, as they affect interactions among humans and other elements of a system [21]. Thus, Iakovidis and Papageorgiou [22] and Kong et al [23] explore methods for modeling human performance to increase understanding of context and domain, including aspects of memory usage, and reasoning. With this approach, they try to bridge some gaps between analysis and the design of health care decision support tools.

Regarding our research question, Iakovidis and Papageorgiou propose the use of fuzzy cognitive mapping, which includes concepts that can be causally interrelated and represent uncertain and imprecise knowledge through fuzzy logic. These concepts encompass tools for modeling and simulation of dynamic systems, based on domain-specific knowledge and experience. The analysis of the domain and cause-effect relations among the system provides additional clues regarding the experts’ knowledge and way of thinking, which increases understanding of work conditions.

Kong et al suggest that the complexity of inference mechanisms and difficulties in representing domain knowledge hamper the design of clinical decision support systems such as the ones used in patient risk assessment. Therefore, representation of human reasoning and uncertain medical knowledge are critical areas that require refined methodologies and techniques.

The paper by Alemdar et al [24] also addresses the challenges in understanding information flow during work performance in order to enable the construction of a health conditions assessment device based on models of machine learning. They also explore the implications of poor understanding of how work is performed in technology design, and its effect on workflows and processes.

Hundt et al [25] highlight that proactive risk assessment methods demand high commitment by team members, and their effectiveness for health information technology implementations has not yet been examined. Although the physician order entry is not a risk assessment process per se, managing patients involves the evaluation of their health conditions and the prioritization of treatment, which is similar to the patient triage process.

### Design Frameworks, Processes, and Methods for Risk Assessment in Health Care

Papers organized in this class of outcomes support the idea that work in health care involves significant information-based cognitive activities; however, it’s mostly supported by exogenously designed information systems. This means that gaps of information about the domain and insufficient input from end users on their needs and practices might bring limitations to the design process.

McClean et al [26] aim at identifying better pathways to patients based on their characteristics such as age, gender, and diagnosis. Therefore, determining the pathway of the patient enables the assessment of patients’ risks.

According to Card et al [27], risk management in health care is largely concerned with routine risks that stem from everyday service provision, which makes it possible for health care organizations to learn from experience and make risk management more effective. However, regarding emergency operations, workers do not often use previous experience to improve risk management processes.

Pennathur et al [28] study situation awareness during diagnosing—starting with the identification of patients’ complaints and laboratory tests results—as the major concern in designing for decision support in patient triage. Understanding the way workers interpret quantitative and qualitative information from patient history, physical conditions, and many other aspects is essential in generating diagnosis and treatment plans. Moreover, there is strong need for understanding the triggering events of medical errors as well as their correlations in order to decrease the probability of occurrence [29].

### Recommendation or Implementation of Improvements in Risk Assessment Work Situations in Health Care

Papers in this class of outcomes demonstrate some approaches that aim at transforming work situations in patient triage. Many approaches could be found such as mathematical programming, resilience engineering, process management, and so on. We highlight the work of Aringhieri et al [30], in which they state that huge amounts of data about health care workers' activities are never used for improving the system performance and the prioritization of resources. Thus, they suggest that modeling, simulation, and mathematical programming can be successfully applied to an emergency service, in order to evaluate its current performance and to provide suggestions to improve the way resources are prioritized.

We also highlight some studies we present in this section that show the differences between the actions of experienced and inexperienced workers as potential object for analysis in order to enable the design of suitable tools for supporting patient triage [31,32]. Understanding human performance and context variables involved in transferring information from junior staff to senior staff—and, eventually, to nursing staff—is an essential aspect in designing work processes in patient triage, as deficiencies in this process may occur because of not only lack of experience but also unavailability of information about patient conditions, poor risk assessment guidelines, communication failures, and lack of consideration to the human, technical, and patient factors involved in this critical process.

Moreover, we find that some authors seek knowledge and understanding into the health care processes and studying patterns through observations carried out jointly by the research teams in order to ensure multidisciplinary perspectives and enable the improvement of work situations and the design of effective support devices [33-35]. We can see similar approach in use for field researches in ergonomics and human factors [17,36-38].

### Analysis of the Effects of New Technologies or Processes to Risk Assessment in Health Care

The 2 papers in this category [39,40] study how human factors enable the analysis of workers’ strategies and workload in patient triage situations. For example, according to Park et al the use of the electronic notes led to an increased workload for residents because of the longer charting times and the shifted responsibility from workers. Moreover, according to Glasgow et al optimal strategy for patient risk mitigation might be identifying risk at the individual level, although minimal knowledge exists on the accuracy of risk assessment with or without decision support tools.

These studies support the claim that the design of technological devices for medical use should not necessarily follow the design adopted by professionals in their current physical notes, as the social nature of clinical work might be hampered if the specific documenting locations, the medium, and the information needed to complete tasks are not properly addressed.

## Discussion

### Principal Findings

Among the 20 papers discarded after full reading, 11 of them did not address the research question. A total of 2 publications were discarded because of low methodological quality according to the aspects we had defined. The 2 databases that presented more search results initially were IEEE Xplore (614 publications) and ScienceDirect (403 publications). However, in the final assessment, more relevant papers were found in the PubMed and ScienceDirect databases. This may suggest that other researchers looking to obtain high-quality papers in the areas of human factors and ergonomics in health care would be best served to approach these sources first.

We believe that the broad range of the ScienceDirect database contributed to a large number of references found, as well as a large number of relevant papers in the final selection. The ScienceDirect database collects publications from diverse fields, from physical sciences and engineering, life sciences, health sciences, and social sciences and humanities. The PubMed database is relatively more specialized, concentrating on publications from the life sciences and biomedical topics—it uses the Medical Subject Headings (MeSH) controlled vocabulary [41].

Our results suggested that there is some interest in the literature in understanding work performance in patient risk assessment. Furthermore, many different approaches have been taken to try and understand the human cognitive work of patient risk assessment. A broad definition of cognitive engineering was applied here, looking for papers that looked at cognition or work processes, and the perspective was broader than more typical cognitive engineering methods. There were more findings in sources specific to medical applications, although some relevant work was still found in engineering and computer science sources.

A total of 2 papers proposed human factors methods for coping with complexity in risk assessment but were not directly applicable to health care and, therefore, discarded. This finding points out the significance of studies about judgment and uncertainty in risk assessment in multiple domains. It also shows that risk assessment in health care presents many opportunities for the use of human factors and ergonomics to improve work situations.

We found that the most selected papers are related to recommendations for improvement (6 publications), decision support tools (4 publications), and design methods (4 publications), while 2 publications explore the effect of new technologies and processes. Recommendations for improvements typically seek transformations in work situations in order to help people work better, more comfortably, mitigating harmful situations, and reducing problems to workers. Studies that examined decision support tools presented the general aspects of developing technology to support decision making in patient triage, such as guidelines, implementation aspects, and milestones in the adoption of decision support tools for patient triage. Design methods refer to techniques, concepts, and modeling tools for coping with complexity.

Some approaches taken by our selected papers related to each other to some extent, especially in developing an understanding of human behavior in complex systems and in finding ways to improve these work situations. For example, some papers presented technologies for patient triage, while discussing how some technologies affect the workload for practitioners. Similarly, design methods were often related to technology as some papers presented design techniques, concepts, and tools that enable the identification of opportunities for information technology or the design of medical decision support. Moreover, opportunities for information technology are, essentially, opportunities for improvement in workflow and practice.

Therefore, the results showed that most related research explored the potential of cognitive engineering to provide tools to improve the design for complex work situations such as risk assessment in health care work environments, although the effects of these applications on human performance have not been extensively assessed.

### Conclusions

This literature review gathered recent contributions to multiple areas, from engineering to biomedical, that cognitive engineering gives for the design of tools for health care risk assessment, especially by contributing knowledge about work performance in such settings. In this paper, we presented information about how this research topic has been approached, results, accomplishments, and opportunities for further research.

Papers selected for review were very diverse in terms of the aims of the study, the underlying theoretical frameworks and methodologies used, reflecting how interdisciplinary our research topic is, and the wide range of research backgrounds employed in finding answers to our research question.

Furthermore, results included studies from several areas such as medicine, engineering, and computer science. We did not present specific research question associated with each area; therefore, some papers might have been excluded for not addressing the research question, although they might have explored our research theme to some extent.

An opportunity for further studies would be to expand the search to include other contributions of human factors and ergonomics to the design for health care—rather than specific contributions to patient risk assessment—as well as the contributions of other areas to the risk assessment in health care. This could address important aspects, for example, which areas have made recent contributions to the improvement of health care services, and subsequently to the risk assessment in health care environments. Moreover, as risk assessment is a topic present in many areas, further research might be interesting to collect studies about the design for risk assessment in other areas rather than health care.

## References

[1] Mackway-Jones K, Marsden J, Windle J (2006). Emergency triage.

[2] Savassi LC, Carvalho HR, Mariano FM, Lamberti CA, Mendonça MF, Yamana GF, Pereira RP (2012). Proposal of a protocol for individual risk classification for home care in primary health. J Manag Prim Health Care.

[3] Lowe RA, Bindman AB, Ulrich SK, Norman G, Scaletta TA, Keane D, Washington D, Grumbach K (1994). Refusing Care to Emergency Department Patients: Evaluation of Published Triage Guidelines. Annals of Emergency Medicine.

[4] Beveridge R, Ducharme J, Janes L, Beaulieu S, Walter S (1999). Reliability of the Canadian Emergency Department Triage and Acuity Scale: Interrater Agreement. Annals of Emergency Medicine.

[5] Cioffi J (1998). Decision making by emergency nurses in triage assessments. Accident and Emergency Nursing.

[6] McCann TV, Clark E, McConnachie S, Harvey I (2007). Deliberate self-harm: emergency department nurses' attitudes, triage and care intentions. J Clin Nurs.

[7] Murdoch J, Barnes R, Pooler J, Lattimer V, Fletcher E, Campbell JL (2015). The impact of using computer decision-support software in primary care nurse-led telephone triage: interactional dilemmas and conversational consequences. Soc Sci Med.

[8] Goldenberg R, Eilot D, Begelman G, Walach E, Ben-Ishai E, Peled N (2012). Computer-aided simple triage (CAST) for coronary CT angiography (CCTA). Int J Comput Assist Radiol Surg.

[9] Nemeth C, Wears C, Woods D, Hollnagel E, Cook R, Henriksen K, Battles JB, Keyes MA (2008). Minding the Gaps: Creating Resilience in Health Care. Advances in Patient Safety: New Directions and Alternative Approaches (Vol. 3: Performance and Tools).

[10] Carayon P, Wetterneck TB, Rivera-Rodriguez AJ, Hundt AS, Hoonakker P, Holden R, Gurses AP (2014). Human factors systems approach to healthcare quality and patient safety. Applied Ergonomics.

[11] Carayon P (2012). Sociotechnical systems approach to healthcare quality and patient safety. Work.

[12] Ottino JM (2004). Engineering complex systems. Nature.

[13] Rasmussen J (1979). On the Structure of Knowledge - A Morphology of Mental Models in a Man-Machine System Context, Risø-M-2192 Technical Report.

[14] Pavard B, Dugdale J, Minai AA, Bar-Yam Y (2006). The Contribution Of Complexity Theory To The Study Of Socio-Technical Cooperative Systems. Unifying Themes in Complex Systems, Vol. IIIB: New Research (New England Complex Systems Institute Series on Complexity).

[15] Hollnagel E, Woods D (2005). Joint cognitive systems: foundations of cognitive systems engineering.

[16] Rasmussen J, Pejtersen AM, Goodstein LP (1994). Cognitive systems engineering.

[17] Theureau J, Hollnagel E (2003). Course-of-action analysis and course-of-action centered design. Handbook of cognitive task design.

[18] Higgins P, Green S (2011). Cochrane handbook for systematic reviews of interventions.

[19] Khan KS, Kunz R, Kleijnen J, Antes G (2003). Five steps to conducting a systematic review. J R Soc Med.

[20] Kitchenham S, Charters S (2007). Guidelines for performing systematic literature reviews in software engineering.

[21] International Ergonomics Association What is Ergonomics.

[22] Iakovidis DK, Papageorgiou E (2011). Intuitionistic fuzzy cognitive maps for medical decision making. IEEE Trans Inf Technol Biomed.

[23] Kong G, Xu D, Body R, Yang J, Mackway-Jones K, Carley S (2012). A belief rule-based decision support system for clinical risk assessment of cardiac chest pain. European Journal of Operational Research.

[24] Alemdar H, Tunca C, Ersoy C (2014). Daily life behaviour monitoring for health assessment using machine learning: bridging the gap between domains. Pers Ubiquit Comput.

[25] Hundt AS, Adams JA, Schmid JA, Musser LM, Walker JM, Wetterneck TB, Douglas SV, Paris BL, Carayon P (2013). Conducting an efficient proactive risk assessment prior to CPOE implementation in an intensive care unit. Int J Med Inform.

[26] McClean S, Barton M, Garg L, Fullerton K (2011). A modeling framework that combines markov models and discrete-event simulation for stroke patient care. ACM Trans. Model. Comput. Simul.

[27] Card AJ, Harrison H, Ward J, Clarkson PJ (2012). Using prospective hazard analysis to assess an active shooter emergency operations plan. J Healthc Risk Manag.

[28] Pennathur PR, Bisantz AM, Fairbanks RJ, Drury CG, Lin L (2014). Following the trail: understanding information flow in the emergency department. Cogn Tech Work.

[29] Cagliano AC, Grimaldi S, Rafele C (2011). A systemic methodology for risk management in healthcare sector. Safety Science.

[30] Aringhieri R, Carello G, Morale D (2013). Supporting decision making to improve the performance of an Italian Emergency Medical Service. Ann Oper Res.

[31] Hepgul N, Kodate N, Anderson JE, Henderson M, Ranjith G, Hotopf M, Pariante CM (2012). Understanding clinical risk decision making regarding development of depression during interferon-alpha treatment for hepatitis-C: a qualitative interview study. Int J Nurs Stud.

[32] Johnston MJ, Arora S, Darzi A (2015). Escalation of Care in Surgery: A Systematic Risk Assessment to Prevent Avoidable Harm in Hospitalized Patients. Ann Surg.

[33] Norris B, West J, Anderson O, Davey G, Brodie A (2014). Taking ergonomics to the bedside--a multi-disciplinary approach to designing safer healthcare. Appl Ergon.

[34] Hastings SN, Whitson HE, Sloane R, Landerman LR, Horney C, Johnson KS (2014). Using the past to predict the future: latent class analysis of patterns of health service use of older adults in the emergency department. J Am Geriatr Soc.

[35] Ferguson E, Starmer C (2013). Incentives, expertise, and medical decisions: testing the robustness of natural frequency framing. Health Psychol.

[36] Wisner A (1995). Understanding problem building: ergonomic work analysis. Ergonomics.

[37] Crandall B, Klein G, Hoffman RL (2006). Crandall, G. Working minds: a practitioner's guide to cognitive task analysis.

[38] Jatobá A, Bellas HC, Bonfatti R, Burns CM, Vidal MC, Carvalho PV (2015). Designing for patient risk assessment in primary health care: a case study for ergonomic work analysis. Cogn Tech Work.

[39] Park SY, Lee SY, Chen Y (2012). The effects of EMR deployment on doctors' work practices: a qualitative study in the emergency department of a teaching hospital. Int J Med Inform.

[40] Glasgow RE, Hawn MT, Hosokawa PW, Henderson WG, Min S, Richman JS, Tomeh MG, Campbell D, Neumayer LA, Ds3 Study Group (2014). Comparison of prospective risk estimates for postoperative complications: human vs computer model. J Am Coll Surg.

[41] Bodenreider O, Nelson SJ, Hole WT, Chang HF (1998). Beyond synonymy: exploiting the UMLS semantics in mapping vocabularies. Proc AMIA Symp.

[42] Reason J (1995). Understanding adverse events: human factors. Qual Health Care.

